# Waist circumference, waist-hip ratio and waist-height ratio percentiles and central obesity among Pakistani children aged five to twelve years

**DOI:** 10.1186/1471-2431-11-105

**Published:** 2011-11-21

**Authors:** Muhammad Umair Mushtaq, Sibgha Gull, Hussain Muhammad Abdullah, Ubeera Shahid, Mushtaq Ahmad Shad, Javed Akram

**Affiliations:** 1 Ubeera Memorial Research Society, Allama Iqbal Medical College, Lahore 54000, Pakistan; 2 District Health Office Nankana Sahib, Punjab Department of Health, Nankana Sahib 39100, Pakistan

## Abstract

**Background:**

Central obesity has been associated with the risk of cardiovascular and metabolic disease in children and anthropometric indices predictive of central obesity include waist circumference (WC), waist-hip ratio (WHR) and waist-height ratio (WHtR). South Asian children have higher body fat distribution in the trunk region but the literature regarding WC and related indices is scarce in this region. The study was aimed to provide age- and gender-specific WC, WHR and WHtR smoothed percentiles, and to explore prevalence and correlates of central obesity, among Pakistani children aged five to twelve years.

**Methods:**

A population-based cross-sectional study was conducted with a representative multistage random cluster sample of 1860 primary school children aged five to twelve years in Lahore, Pakistan. Smoothed percentile curves were constructed for WC, WHR and WHtR by the LMS method. Central obesity was defined as having both age- and gender-specific WC percentile ≥90^th ^and WHtR ≥0.5. Chi-square test was used as the test of trend. Multivariate logistic regression was used to quantify the independent predictors of central obesity and adjusted odds ratios (aOR) with 95% CI were obtained. Linear regression was used to explore the independent determinants of WC and WHtR. Statistical significance was considered at P < 0.05.

**Results:**

First ever age- and gender-specific smoothed WC, WHR and WHtR reference curves for Pakistani children aged five to twelve years are presented. WC increased with age among both boys and girls. Fiftieth WC percentile curves for Pakistani children were higher as compared to those for Hong Kong and British children, and were lower as compared to those for Iranian, German and Swiss children. WHR showed a plateau pattern among boys while plateau among girls until nine years of age and decreased afterwards. WHtR was age-independent among both boys and girls, and WHtR cut-off of ≥0.5 for defining central obesity corresponded to 85^th ^WHtR percentile irrespective of age and gender. Twelve percent children (95% CI 10.1-13.0) had a WC ≥90^th ^percentile and 16.5% children (95% CI 14.7-18.1) had a WHtR ≥0.5 while 11% children (95% CI 8.9-11.6) had both WC ≥90^th ^percentile and WHtR ≥0.5. Significant predictors of central obesity included higher grade, urban area with high socioeconomic status (SES), high-income neighborhood and higher parental education. Children studying in higher grade (aOR 5.11, 95% CI 1.76-14.85) and those living in urban area with high SES (aOR 82.34, 95% CI 15.76-430.31) showed a significant independent association. Urban area with high SES and higher parental education showed a significant independent association with higher WC and higher WHtR while higher grade showed a significant independent association with higher WC.

**Conclusions:**

Comprehensive worldwide reference values are needed to define central obesity and the present study is the first one to report anthropometric indices predictive of central obesity for Pakistani school-aged children. Eleven percent children were centrally obese and strong predictors included higher grade, urban area with high SES and higher parental education. These findings support the need for developing a National strategy for childhood obesity and implementing targeted interventions, prioritizing the higher social class and involving communities.

## Background

Obesity is a global epidemic and children are the worst affected with an estimated ten percent of school-aged children being overweight and one quarter of these being obese worldwide [1]. Childhood obesity epidemic is now penetrating the developing countries including Pakistan, especially in the affluent urban population [2,3]. Central obesity has been associated with the risk of cardiovascular and metabolic disease in children and anthropometric indices predictive of childhood central obesity include waist circumference (WC), waist-hip ratio (WHR) and waist-height ratio (WHtR) [4-8]. However, measurement of WC is not yet a common practice in children. Most of the studies reporting percentiles for WC and related indices in school-aged children were conducted in the developed countries including United States, Australia and European region [9-19]. In Asia, WC percentiles have been reported for Middle-eastern and Southeast Asian children [20-24]. South Asian children have higher body fat distribution in the trunk region [25-29]; however, the data on WC and related indices is scarce in this region. Comprehensive worldwide reference values for WC and related indices are needed to establish an internationally accepted age-, gender-, and ethnicity-specific definition of central obesity in children. The study was aimed to provide first ever age- and gender-specific WC, WHR and WHtR smoothed percentiles, and to explore prevalence and correlates of central obesity, among Pakistani children aged five to twelve years.

## Methods

### Design, setting and sample

A population-based cross-sectional study, the Nutritional Assessment among School-going Children in Lahore, Pakistan (NASCL study), was conducted among primary school children aged five to twelve years in 2009-10. Lahore is the capital of Pakistan's most populous province Punjab and a metropolis with multiethnic populations. It has a population of nine million, including 2.5 million primary school children, and 81% of the population resides in urban area (Administrative data, Government of Punjab, 2010).

A stratified multistage random cluster sample of 1860 children aged five to twelve years in twelve primary schools of City District Lahore was enrolled. Stratified sampling, based on the population and educational system characteristics, was used to have proportionate representation of gender, area of residence and socioeconomic status (SES). About 1200 public and 1100 private primary schools in Lahore registered with the Punjab Department of Education were listed. The listed schools were stratified according to the geographic area and monthly fee structure of schools into following four strata: a) urban with high SES (urban area and fee > 2500 PKR), b) urban with middle SES (urban area and fee = 1000-2500 PKR), c) urban with low SES (urban area and fee < 1000 PKR), and d) rural with low/disadvantaged SES (rural area and fee ~100 PKR or free). The former two strata included private (including public-private mix) schools and the later two strata included public schools. In Pakistan, public schools cater low SES urban and rural children while high and middle SES urban children are educated in private and public-private mix schools. Three schools were selected at random from each stratum and contacted by the Departments of Education and Health to participate voluntarily in the study. If school administration refused to participate, next school was selected randomly from the respective stratum. For each school, a list of all classes in five grades (one to five) was obtained and one class in each grade was selected at random. In this way, sixty classes, five from each school, were selected. For each of the selected classes, first thirty-one children on class attendance register, present on data collection day and aged five to twelve years, were included in the study. Children suffering from any known metabolic syndrome (like Prader-Willi syndrome) and those not willing to participate in the study were excluded. Sample size was calculated using Epi Info 6.04d (United States' Centers for Disease Control and Prevention, 2004) with a confidence (1-α) of 95%, anticipated prevalence of 5% and margin of error of ± 1. The minimum sample size calculated was 1823 and a sample of 1860 was deemed sufficient.

### Data Collection

The sampled schools were visited on pre-arranged dates in summer 2009 by a team of trained senior medical students lead by the Principal Investigator. Health education of children and teachers was also carried out after data collection in the respective school. Height was measured by portable stadiometers that were standardized and calibrated before the measurements. Feet were placed together with heels, buttocks and shoulder blades against the stick and head was positioned in the Frankfurt horizontal plane. WC and hip circumference (HC) were measured according to the World Health Organization (WHO) recommendations [30,31]. The subject was asked to stand relaxed with arms at the sides, feet positioned close together and weight evenly distributed across feet. WC was measured midway between the lowest rib and the superior border of iliac crest at the end of normal expiration with a stretch-resistant measuring tape positioned at a level parallel to the floor. HC was measured at the level of widest portion of buttocks (trochanters). All measurements were in centimeters (cm) to the nearest 0.1 cm. All children were measured in mornings or early afternoons without shoes and in light summer school-uniform.

Demographic information of all officially enrolled students in the sampled classes was obtained before data collection that included gender, date of birth, residential address and parental education. If demographic information of students was not found on official rosters, it was obtained from the classroom teachers. Parental education level was based on the parent with the highest total years of schooling and neighborhood income level was based on the approximate income estimate of child's residential area obtained from the Revenue Department of City District Government Lahore. Quality control measures and good practices including training, pre-testing the processes and materials, field monitoring of data collection, logistics management and daily meetings of the study teams were ensured. Informed consent statement was printed on the study form. Verbal informed consent for the child to participate in the study was taken from class teachers and school heads. As the study involved no invasive procedure, verbal informed consent was deemed sufficient. The study was approved by the Ethical Review Board of Allama Iqbal Medical College, Lahore. Permissions to conduct the survey were granted by the Punjab Departments of Education and Health, and the sampled schools.

### Statistical Analysis

Data were entered and analyzed by manual and computerized checking using SPSS version 18.0 (SPSS Inc. Chicago IL, United States, 2009). Age was calculated to the precise day by subtracting the date of birth from the date of examination. Smoothed age- and gender-specific percentile curves were constructed for WC, WHR and WHtR by the LMS method [32]. Fiftieth percentile curves for WC were compared with the previous studies in Hong Kong, United Kingdom, Iran, Germany, Switzerland and China that measured WC at the same site. Cut-off of ≥90^th ^age- and gender-specific percentile for WC [15,17,18,24,33,34] and ≥0.5 for WHtR [15,35-37] had been suggested for defining central obesity. Central obesity was defined as having both age- and gender-specific WC percentile ≥90^th ^and WHtR ≥0.5. Bivariate analysis, using chi-square test as the test of trend, was conducted to compare the prevalence of central obesity among the study variables. Multivariate logistic regression was used to quantify the independent predictors of central obesity and adjusted odds ratios (aOR) with 95% confidence interval (CI) were obtained. Linear regression was used to explore the predictive power of all socio-demographic factors significantly associated with central obesity (as independent variables) in relation to WC and WHtR (as dependent variables). Statistical significance was considered at P < 0.05 and all tests were two-sided.

## Results

The study included a sample of 1860 primary school children aged five to twelve years. The male-female ratio was 1.11 with 52.5% boys and 47.5% girls. The sample involved 20% children from each grade and 25% children from each area and SES stratum. Seventy-five percent children were urban and 25% were rural. The median age (range) was 101.2 (60-154) months. The mean and standard deviation (SD) for WC, HC, height, WHR and WHtR were 58.4 (7.9) cm, 67.2 (11.4) cm, 128.4 (11.4) cm, 0.88 (0.17) and 0.46 (0.05) respectively. Age- and gender-specific mean (SD) WC, WHR and WHtR are presented in Table 1.

**Table 1:** Mean and standard deviation (SD) for waist circumference (WC), waist-hip ratio (WHR) and waist-height ratio (WHtR) of Pakistani primary school children aged five to twelve years (n = 1860)

	n	WC (cm)	WHR	WHtR
**Boys (n = 977)**				
5 years (61-71 months)	84	54.0 (5.8)	0.89 (0.04)	0.46 (0.04)
6 years (72-83 months)	161	54.0 (5.6)	0.89 (0.04)	0.46 (0.04)
7 years (84-95 months)	160	55.8 (5.8)	0.88 (0.04)	0.45 (0.05)
8 years (96-107 months)	158	58.3 (6.6)	0.88 (0.04)	0.45 (0.04)
9 years (108-119 months)	161	60.8 (7.9)	0.88 (0.05)	0.45 (0.05)
10 years (120-131 months)	147	62.7 (9.8)	0.88 (0.05)	0.45 (0.06)
11 years (132-143 months)	69	62.0 (7.2)	0.88 (0.06)	0.45 (0.05)
12 years (144-155 months)	37	61.2 (5.6)	0.87 (0.04)	0.44 (0.03)
**Girls (n = 883)**				
5 years (61-71 months)	72	52.6 (3.4)	0.86 (0.05)	0.46 (0.03)
6 years (72-83 months)	143	54.4 (6.0)	0.85 (0.05)	0.46 (0.04)
7 years (84-95 months)	157	57.0 (7.2)	0.87 (0.08)	0.46 (0.05)
8 years (96-107 months)	159	58.7 (7.0)	0.86 (0.06)	0.46 (0.05)
9 years (108-119 months)	151	61.6 (8.3)	0.86 (0.06)	0.46 (0.06)
10 years (120-131 months)	120	62.3 (8.9)	0.84 (0.06	0.45 (0.06)
11 years (132-143 months)	62	62.3 (8.4)	0.80 (0.06)	0.43 (0.05)
12 years (144-155 months)	19	62.6 (7.9)	0.81 (0.05)	0.43 (0.04)

**Figure 1:**
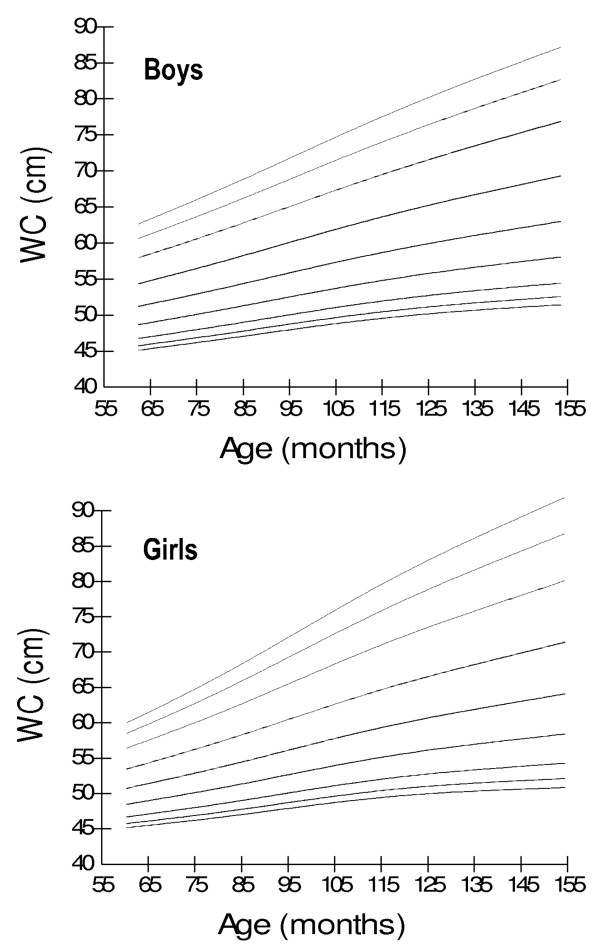
**Smoothed waist circumference (WC) percentile curves for Pakistani primary school boys (n = 997) and girls (n = 883) aged five to twelve years**.

**Figure 2:**
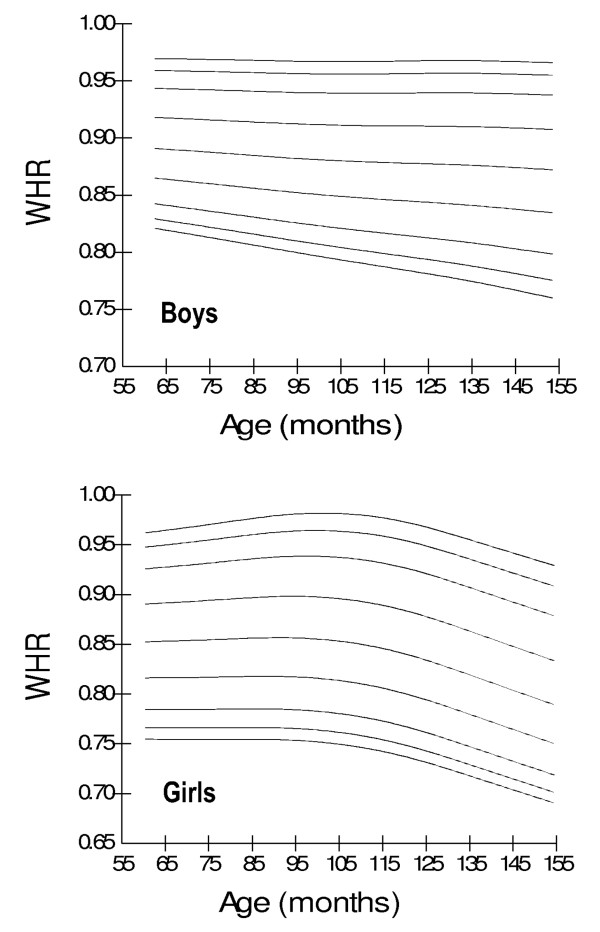
**Smoothed waist-hip ratio (WHR) percentile curves for Pakistani primary school boys (n = 997) and girls (n = 883) aged five to twelve years**.

**Figure 3:**
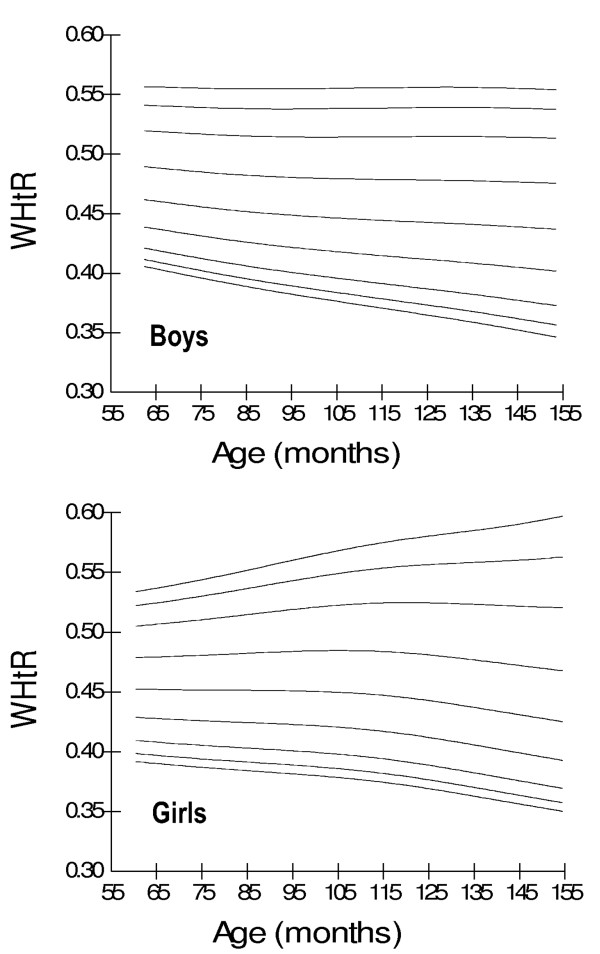
**Smoothed waist-height ratio (WHtR) percentile curves for Pakistani primary school boys (n = 997) and girls (n = 883) aged five to twelve years**.

**Figure 4:**
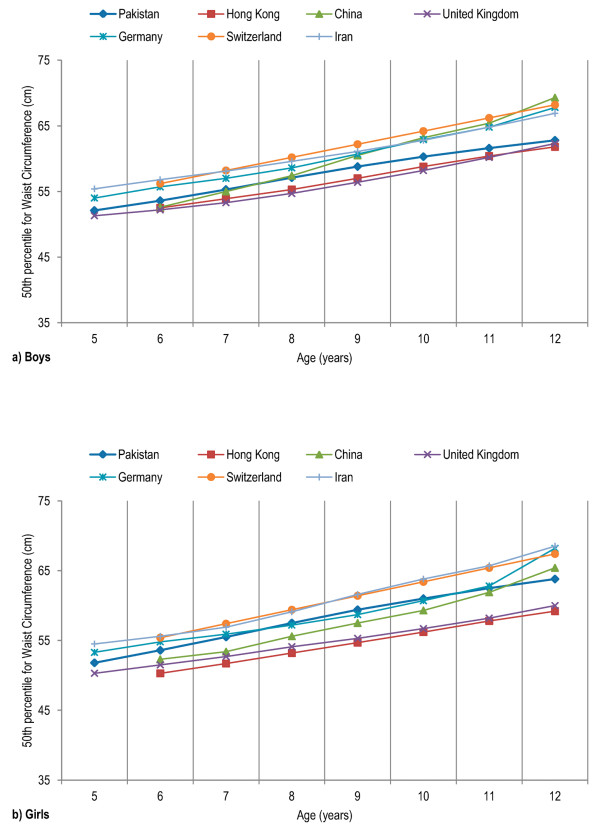
**Comparison of fiftieth waist circumference (WC) percentile curves for Pakistani primary school boys (a) and girls (b) aged five to twelve years with previous studies that measured WC at the same site**. WC was measured midway between the lowest rib and the superior border of iliac crest, in accordance with the WHO recommendations.

Age- and gender-specific WC percentiles [Table 2] [Figure 1], age- and gender-specific WHR percentiles [Table 3] [Figure 2], and age- and gender-specific WHtR percentiles [Table 4] [Figure 3**] **were developed and smoothed by the LMS method. WC increased with age among both boys and girls. Fiftieth WC percentile curves for Pakistani children were higher as compared to those for Hong Kong and British children, and were lower as compared to those for Iranian, German and Swiss children [Figure 4]. Fiftieth percentile curves for Chinese children were lower than the study sample in younger age groups and were higher in older children. WHR showed a plateau pattern among boys while plateau among girls until nine years of age and decreased afterwards. WHtR was age-independent among both boys and girls with a mean difference of 0.02. WHtR cut-off of ≥0.5 for defining central obesity corresponded to 85^th ^WHtR percentile irrespective of age and gender with a mean difference of < 0.01 [Table 5].

**Table 2:** Age- and gender-specific smoothed waist circumference (WC) percentiles for Pakistani primary school children aged five to twelve years (n = 1860)

	Percentiles								
	
	**3**^**rd**^	**5**^**th**^	**10**^**th**^	**25**^**th**^	**50**^**th**^	**75**^**th**^	**90**^**th**^	**95**^**th**^	**97**^**th**^
**Boys (n = 977)**									
5 years (61-71 months)	45.7	46.4	47.4	49.4	52.1	55.3	59.1	61.9	64.1
6 years (72-83 months)	46.7	47.4	48.5	50.7	53.6	57.2	61.5	64.6	67.1
7 years (84-95 months)	47.7	48.5	49.7	52.1	55.3	59.4	64.1	67.7	70.5
8 years (96-107 months)	48.8	49.6	51.0	53.6	57.1	61.6	66.9	70.9	74.0
9 years (108-119 months)	49.7	50.6	52.1	54.9	58.8	63.7	69.5	74.0	77.5
10 years (120-131 months)	50.5	51.4	53.0	56.1	60.3	65.6	72.0	76.9	80.7
11 years (132-143 months)	51.1	52.1	53.8	57.1	61.6	67.4	74.2	79.5	83.6
12 years (144-155 months)	51.5	52.6	54.4	58.0	62.8	68.9	76.2	81.9	86.2
**Girls (n = 883)**									
5 years (61-71 months)	45.8	46.4	47.4	49.3	51.8	54.7	58.0	60.4	62.1
6 years (72-83 months)	46.7	47.4	48.5	50.7	53.6	57.1	61.0	63.9	66.0
7 years (84-95 months)	47.7	48.5	49.8	52.2	55.5	59.6	64.3	67.7	70.4
8 years (96-107 months)	48.7	49.6	51.0	53.8	57.5	62.2	67.6	71.8	74.9
9 years (108-119 months)	49.6	50.5	52.2	55.2	59.4	64.7	70.9	75.7	79.4
10 years (120-131 months)	50.2	51.3	53.0	56.4	61.0	66.9	74.0	79.4	83.6
11 years (132-143 months)	50.6	51.8	53.7	57.4	62.5	69.0	76.8	82.8	87.4
12 years (144-155 months)	50.9	52.2	54.3	58.3	63.8	70.9	79.4	85.9	91.0

**Table 3:** Age- and gender-specific smoothed waist-hip ratio (WHR) percentiles for Pakistani primary school children aged five to twelve years (n = 1860)

	Percentiles								
	
	**3**^**rd**^	**5**^**th**^	**10**^**th**^	**25**^**th**^	**50**^**th**^	**75**^**th**^	**90**^**th**^	**95**^**th**^	**97**^**th**^
**Boys (n = 977)**									
5 years (61-71 months)	0.82	0.83	0.84	0.86	0.89	0.92	0.94	0.96	0.97
6 years (72-83 months)	0.81	0.82	0.84	0.86	0.89	0.92	0.94	0.96	0.97
7 years (84-95 months)	0.80	0.81	0.83	0.86	0.88	0.91	0.94	0.96	0.97
8 years (96-107 months)	0.80	0.81	0.82	0.85	0.88	0.91	0.94	0.96	0.97
9 years (108-119 months)	0.79	0.80	0.82	0.85	0.88	0.91	0.94	0.96	0.97
10 years (120-131 months)	0.78	0.79	0.81	0.84	0.88	0.91	0.94	0.96	0.97
11 years (132-143 months)	0.77	0.79	0.81	0.84	0.88	0.91	0.94	0.96	0.97
12 years (144-155 months)	0.77	0.78	0.80	0.84	0.87	0.91	0.94	0.96	0.97
**Girls (n = 883)**									
5 years (61-71 months)	0.76	0.77	0.79	0.82	0.85	0.89	0.93	0.95	0.97
6 years (72-83 months)	0.76	0.77	0.79	0.82	0.86	0.9	0.93	0.96	0.97
7 years (84-95 months)	0.76	0.77	0.79	0.82	0.86	0.9	0.94	0.96	0.98
8 years (96-107 months)	0.75	0.76	0.78	0.82	0.86	0.9	0.94	0.97	0.98
9 years (108-119 months)	0.75	0.76	0.78	0.81	0.85	0.89	0.93	0.96	0.98
10 years (120-131 months)	0.73	0.74	0.76	0.79	0.83	0.88	0.92	0.95	0.97
11 years (132-143 months)	0.72	0.73	0.74	0.78	0.82	0.86	0.90	0.93	0.95
12 years (144-155 months)	0.70	0.71	0.73	0.76	0.80	0.84	0.89	0.92	0.94

**Table 4:** Age- and gender-specific smoothed waist-height ratio (WHtR) percentiles for Pakistani primary school children aged five to twelve years (n = 1860)

	Percentiles								
	
	**3**^**rd**^	**5**^**th**^	**10**^**th**^	**25**^**th**^	**50**^**th**^	**75**^**th**^	**90**^**th**^	**95**^**th**^	**97**^**th**^
**Boys (n = 977)**									
5 years (61-71 months)	0.40	0.41	0.42	0.44	0.46	0.49	0.52	0.54	0.56
6 years (72-83 months)	0.40	0.40	0.41	0.43	0.46	0.49	0.52	0.54	0.56
7 years (84-95 months)	0.39	0.39	0.40	0.43	0.45	0.48	0.52	0.54	0.56
8 years (96-107 months)	0.38	0.39	0.40	0.42	0.45	0.48	0.52	0.54	0.56
9 years (108-119 months)	0.37	0.38	0.39	0.42	0.45	0.48	0.52	0.54	0.56
10 years (120-131 months)	0.37	0.37	0.39	0.41	0.44	0.48	0.52	0.54	0.56
11 years (132-143 months)	0.36	0.37	0.38	0.41	0.44	0.48	0.52	0.54	0.56
12 years (144-155 months)	0.35	0.36	0.38	0.40	0.44	0.48	0.52	0.54	0.56
**Girls (n = 883)**									
5 years (61-71 months)	0.39	0.40	0.41	0.43	0.45	0.48	0.51	0.53	0.54
6 years (72-83 months)	0.39	0.39	0.41	0.43	0.45	0.48	0.51	0.53	0.55
7 years (84-95 months)	0.38	0.39	0.40	0.43	0.45	0.48	0.52	0.54	0.56
8 years (96-107 months)	0.38	0.39	0.40	0.42	0.45	0.49	0.52	0.55	0.57
9 years (108-119 months)	0.38	0.38	0.40	0.42	0.45	0.49	0.53	0.55	0.58
10 years (120-131 months)	0.37	0.38	0.39	0.41	0.44	0.48	0.53	0.56	0.58
11 years (132-143 months)	0.36	0.37	0.38	0.41	0.44	0.48	0.53	0.56	0.59
12 years (144-155 months)	0.35	0.36	0.37	0.40	0.43	0.47	0.52	0.56	0.60

**Table 5:** Age- and gender-specific eighty-fifth percentile values for waist-height ratio (WHtR) corresponding to cut-off value of 0.5 for defining central obesity among Pakistani primary school children aged five to twelve years (n = 1860)

Age	Boys (n = 997)	Girls (n = 883)
5 years (61-71 months)	0.507	0.499
6 years (72-83 months)	0.504	0.500
7 years (84-95 months)	0.502	0.504
8 years (96-107 months)	0.501	0.507
9 years (108-119 months)	0.501	0.508
10 years (120-131 months)	0.501	0.507
11 years (132-143 months)	0.501	0.504
12 years (144-155 months)	0.500	0.500

Twelve percent children (95% CI 10.1-13.0) had a WC ≥90^th ^percentile and 16.5% children (95% CI 14.7-18.1) had a WHtR ≥0.5 while 11% children (95% CI 8.9-11.6) had both WC ≥90^th ^percentile and WHtR ≥0.5. Prevalence of central obesity significantly increased with grade among both boys and girls [Table 6]. Higher grade, urban area with high SES, high-income neighborhood and higher parental education were significantly associated with central obesity (all P < 0.001) [Table 7]. Multivariate logistic regression analysis was adjusted simultaneously for socio-demographic factors significantly associated with central obesity and controlled for age and gender. Significant independent predictors of central obesity included higher grade (aOR 5.11, 95% CI 1.76-14.85) and urban area with high SES (aOR 82.34, 95% CI 15.76-430.31) [Table 8]. Linear regression analyses were adjusted for socio-demographic factors significantly associated with central obesity and controlled for age and gender. Higher grade, urban area with high SES and higher parental education showed a significant independent association with higher WC [Table 9]. Urban area with high SES and higher parental education showed a significant independent association with higher WHtR [Table 10].

**Table 6:** Grade- and Gender-specific prevalence of central obesity among Pakistani primary school children aged five to twelve years (n = 1860)

	WC ≥90^th ^percentile		WHtR ≥0.5		**Centrally Obese**^**a**^	
	(n = 213)		(n = 304)		(n = 189)	
	
	n (%)	P Value	n (%)	P Value	n (%)	P Value
**Boys (n = 997)**	**103 (10.5)**		**156 (16.2)**		**93 (9.5)**	
Grade one (n = 233)	19 (8.2)	0.054	50 (21.5)	0.026	16 (6.9)	0.033
Grade two (n = 187)	13 (7.0)		23 (12.3)		11 (5.9)	
Grade three (n = 183)	18 (9.8)		23 (12.6)		17 (9.3)	
Grade four (n = 184)	26 (14.1)		35 (19.0)		25 (13.6)	
Grade five (n = 190)	27 (14.2)		25 (13.2)		24 (12.6)	
**Girls (n = 883)**	**148 (16.8)**		**110 (12.5)**		**96 (10.9)**	
Grade one (n = 139)	16 (11.5)	0.004	20 (14.4)	< 0.001	13 (9.4)	0.001
Grade two (n = 185)	11 (5.9)		15 (8.1)		7 (3.8)	
Grade three (n = 189)	31 (16.4)		41 (21.7)		28 (14.8)	
Grade four (n = 188)	33 (17.6)		45 (23.9)		30 (16.0)	
Grade five (n = 182)	19 (10.4)		27 (14.8)		18 (9.9)	

^a^≥90^th ^age- and gender-specific waist circumference (WC) percentile and ≥0.5 waist-height ratio (WHtR)

**Table 7:** Socio-demographic correlates of central obesity among Pakistani primary school children aged five to twelve years (n = 1860)

	Total Sample (n = 1860)	Centrally Obese^a ^(n = 189)	
	
Characteristics	n (%)	n (%)	P Value
**Gender**			
Boys	977 (52.5)	93 (9.5)	0.335
Girls	883 (47.5)	96 (10.9)	
**Grade**			
One	372 (20.0)	29 (7.8)	< 0.001
Two	372 (20.0)	18 (4.8)	
Three	372 (20.0)	45 (12.1)	
Four	372 (20.0)	55 (14.8)	
Five	372 (20.0)	42 (11.3)	
**Area and socioeconomic status (SES)**			
Urban with high SES	465 (25.0)	101 (21.7)	< 0.001
Urban with middle SES	465 (25.0)	58 (12.5)	
Urban with low SES	465 (25.0)	28 (6.0)	
Rural (low/disadvantaged SES)	465 (25.0)	2 (0.4)	
**Neighborhood income**			
Low	651 (35.0)	27 (4.1)	< 0.001
Middle	910 (48.9)	97 (10.7)	
High	299 (16.1)	65 (21.7)	
**Parental education**			
Illiterate	366 (19.7)	6 (1.6)	< 0.001
High school	496 (26.7)	25 (5.0)	
College	531 (28.5)	71 (13.4)	
Higher education	467 (25.1)	87 (18.6)	

^a^≥90^th ^age- and gender-specific waist circumference (WC) percentile and ≥0.5 waist-height ratio (WHtR)

**Table 8:** Logistic regression analysis of socio-demographic factors associated with central obesity among Pakistani primary school children aged five to twelve years (n = 1860)^a, b^

Characteristics	Crude OR (95% CI)	P Value	Adjusted OR (95% CI)	P Value
**Grade**				
One	Reference	-	Reference	-
Two	0.70 (0.37-1.31)	0.259	0.80 (0.41-1.56)	0.515
Three	2.29 (1.24-4.22)	0.008	3.15 (1.54-6.45)	0.002
Four	3.36 (1.66-6.80)	0.001	5.47 (2.25-13.31)	< 0.001
Five	2.84 (1.23-6.54)	0.014	5.11 (1.76-14.85)	0.003
**Area and socioeconomic status (SES)**				
Urban with high SES	64.24 (15.74-262.13)	< 0.001	82.34 (15.76-430.31)	< 0.001
Urban with middle SES	32.99 (8.01-135.92)	< 0.001	35.67 (6.95-183.18)	< 0.001
Urban with low SES	14.83 (3.51-62.64)	< 0.001	13.67 (2.86-65.40)	0.001
Rural (low/disadvantaged SES)	Reference	-	Reference	-
**Neighborhood income**				
Low	0.16 (0.10-0.25)	< 0.001	1.50 (0.77-2.93)	0.239
Middle	0.43 (0.30-0.61)	< 0.001	0.77 (0.51-1.17)	0.220
High	Reference	-	Reference	-
**Parental education**				
Illiterate	Reference	-	Reference	-
High school	3.37 (1.36-8.31)	0.008	0.73 (0.26-2.03)	0.545
College	9.93 (4.25-23.20)	< 0.001	1.04 (0.37-2.91)	0.938
Higher education	14.13 (6.09-32.77)	< 0.001	1.32 (0.47-3.73)	0.599

^a^The model is controlled for age and gender

^b^≥90^th ^age- and gender-specific waist circumference (WC) percentile and ≥0.5 waist-height ratio (WHtR)

**Table 9:** Linear regression analysis of socio-demographic factors with waist circumference (WC) among Pakistani primary school children aged five to twelve years (n = 1860)^a, b^

Characteristics	Regression coefficient (95% CI)	Standard error	P value
Grade	1.099 (0.686 to 1.511)	0.210	< 0.001
Area and SES^c^	-1.896 (-2.360 to -1.431)	0.237	< 0.001
Higher neighborhood income	-0.049 (-0.690 to 0.591)	0.326	0.880
Higher parental education	0.428 (0.018 to 0.839)	0.209	0.041

^a^The model is controlled for age and gender

^b^R^2 ^for model = 0.280

^c^Area and SES strata: 1. Urban with high SES, 2. Urban with middle SES, 3. Urban with low SES, and 4. Rural with low/disadvantaged SES

**Table 10:** Linear regression analysis of socio-demographic factors with waist-height ratio (WHtR) among Pakistani primary school children aged five to twelve years (n = 1860)^a, b^

Characteristics	Regression coefficient (95% CI)	Standard error	P value
Grade	-0.001 (-0.004 to 0.002)	0.001	0.428
Area and SES^c^	-0.010 (-0.013 to -0.006)	0.002	< 0.001
Higher neighborhood income	-0.004 (-0.009 to 0.001)	0.002	0.056
Higher parental education	0.004 (0.001 to 0.007)	0.001	0.012

^a^The model is controlled for age and gender

^b^R^2 ^for model = 0.068

^c^Area and SES strata: 1. Urban with high SES, 2. Urban with middle SES, 3. Urban with low SES, and 4. Rural with low/disadvantaged SES

## Discussion

First ever age- and gender-specific smoothed percentiles for WC, WHR and WHtR developed from a representative sample of 1860 Pakistani primary school children aged five to twelve years are presented. These charts are based on WC measured midway between the lowest rib and the superior border of iliac crest, in accordance with the WHO recommendations [31].

WC increased with age among both boys and girls, consistent with previous literature [9-24]. There was no gender disparity in WC values, in line with previous findings in children aged five to twelve years [11]. Fiftieth WC percentile curves for Pakistani children were higher as compared to those for Hong Kong and British children, and were lower as compared to those for Iranian, German and Swiss children. WC remains the simplest clinical measure of childhood central obesity. It has been proved a strong predictor of cardiovascular and metabolic disease risk in children [38-47]. It provides a better estimate of visceral adipose tissue than body mass index (BMI) and is significantly more efficient than BMI in predicting insulin resistance, blood pressure, and serum cholesterol and triglyceride levels [38-41]. WHR showed a plateau pattern among boys while remained stable among girls until nine years of age and decreased afterwards, consistent with previous studies [48]. WHR has been used to describe body fat distribution in adults; however, it is influenced by several other body factors and is a poor measure of body fat distribution and risk of related diseases in children [49,50]. WHtR was age-independent among both boys and girls, and suggested WHtR cut-off of ≥0.5 for defining central obesity corresponded to 85^th ^WHtR percentile irrespective of age and gender, in line with previous literature [15,35-37]. Previous studies have strongly correlated WHtR to the risk of cardiovascular and metabolic disease in children, and it has been proposed as an alternative measure for assessing central fatness in children especially for pediatric primary care practice and epidemiological studies, as it is a relatively age-independent measure [8,33,51-56].

Eleven percent of children were centrally obese. Central obesity showed a significantly increasing trend with grade among both boys and girls and higher grade independently predicted the risk of being centrally obese and having higher WC. These findings are consistent with previous studies reporting higher obesity prevalence in older children [57-59]. Urban area with high SES, high-income neighborhood and higher parental education significantly correlated with central obesity. Living in urban area with high SES was a strong independent predictor of being at risk of central obesity. Urban area with high SES and higher parental education were independently associated with higher WC and higher WHtR. Increasing trend in childhood obesity with urbanization had been reported in both the developing and the developed countries [57,60-62]. Association of childhood obesity with high SES and higher parental education had been observed in the developing countries [3,63-68]. However, studies in the developed countries had shown inverse association of SES, neighborhood income level and parental education with obesity [61,69-73]. Different socio-cultural circumstances in the developing countries undergoing nutrition transition explain the contradiction. Association between socioeconomic factors and over-nutrition vary in societies at different stages of nutrition transition. Obesity is positively associated with socioeconomic factors in Asia but not in Latin America [74]. Changes in lifestyle with urbanization including reduced physical activity, increased sedentary living and unhealthy diets, reinforced by many of the cultural changes associated with globalization, are the probable underlying causes [60,75,76]. Efforts to stop childhood obesity should be made on all fronts and targeted interventions, tailored to local circumstances and involving communities, should begin early in life [77].

Variability in the data ascertainment may have introduced an error into the estimates; however, we do not anticipate large or systematic differences. The effects of puberty on anthropometric indices were not explored in the present study; however, future studies are suggested in this regard. Cross-sectional nature of the study should be considered when interpreting the findings reported and future longitudinal studies are warranted to establish the temporal nature and causality of these associations. These findings can be generalized to South Asian primary school children that share the same genetic and environmental factors with the sample.

## Conclusions

Comprehensive worldwide reference values are needed to establish an internationally accepted age-, gender-, and ethnicity-specific definition of central obesity. The present study is the first one to report anthropometric indices predictive of central obesity for Pakistani school-aged children. Eleven percent children were centrally obese and strong predictors included higher grade, urban area with high SES and higher parental education. These findings support the need for developing a National strategy for childhood obesity and implementing targeted interventions, prioritizing the higher social class and involving communities.

## Competing interests

The authors declare that they have no competing interests.

## Authors' contributions

MUM, principal investigator, conceived and implemented the study, analyzed and interpreted the data, prepared the manuscript and supervised the entire project. SG and HMA contributed to the study analysis, interpretation and manuscript preparation. US contributed to the study conception, implementation and analysis. MAS and JA oversaw the study conception, implementation and manuscript preparation. All authors read and approved the final manuscript.

## Pre-publication history

The pre-publication history for this paper can be accessed here:

http://www.biomedcentral.com/1471-2431/11/105/prepub
